# S100B Up-Regulates Macrophage Production of IL1β and CCL22 and Influences Severity of Retinal Inflammation

**DOI:** 10.1371/journal.pone.0132688

**Published:** 2015-07-23

**Authors:** Jennifer Niven, Joseph Hoare, Debbie McGowan, Gayathri Devarajan, Shigeyoshi Itohara, Monique Gannagé, Peter Teismann, Isabel Crane

**Affiliations:** 1 Division of Applied Medicine, University of Aberdeen Institute of Medical Sciences, Foresterhill, Aberdeen, Scotland, United Kingdom; 2 RIKEN Brain Science Institute, Wako, Japan; 3 Division of Rheumatology and Department of Pathology and Immunology, School of Medicine, University of Geneva, Geneva, Switzerland; University of Florida, UNITED STATES

## Abstract

S100B is a Ca^2+^ binding protein and is typically associated with brain and CNS disorders. However, the role of S100B in an inflammatory situation is not clear. The aim of the study was to determine whether S100B is likely to influence inflammation through its effect on macrophages. A murine macrophage cell line (RAW 264.7) and primary bone marrow derived macrophages were used for *in vitro* studies and a model of retinal inflammatory disease in which pathogenesis is highly dependent on macrophage infiltration, Experimental Autoimmune Uveoretinitis, for *in vitro* study. Experimental Autoimmune Uveoretinitis is a model for the human disease posterior endogenous uveoretinitis, a potentially blinding condition, with an autoimmune aetiology, that mainly affects the working age group. To date the involvement of S100B in autoimmune uveoretinitis has not been investigated. Real-time PCR array analysis on RAW 246.7 cells indicated up-regulation of gene expression for various cytokines/chemokines in response to S100B, IL-1β and CCL22 in particular and this was confirmed by real-time PCR. In addition flow cytometry and ELISA confirmed up-regulation of protein production in response to S100B for pro-IL-1β and CCL22 respectively. This was the case for both RAW 264.7 cells and bone marrow derived macrophages. Induction of EAU with retinal antigen in mice in which *S100B* had been deleted resulted in a significantly reduced level of disease compared to wild-type mice, as determined by topical endoscopic fundus imaging and histology grading. Macrophage infiltration was also significantly reduced in *S100B* deleted mice. Real-time PCR analysis indicated that this was associated with reduction in CCL22 and IL-1β in retinas from *S100B* knock-out mice. In conclusion S100B augments the inflammatory response in uveoretinitis and this is likely to be, at least in part, via a direct effect on macrophages.

## Introduction

S100B is a Ca^2+^ binding protein which is abundantly and constitutively expressed in the brain by astrocytes where it has both autocrine and paracrine effects on neurons and glia [1]. To a lesser extent it is also produced by other cell types such as monocytes, macrophages, microglia and T cells [2]. It has both intracellular and extracellular functions [3]. Intracellular S100B is involved in cytoskeletal interactions, Ca^2+^ homeostasis and regulation of enzyme activity [3]. S100B can also be secreted and extracellular activities are less clear cut and may depend on concentration. At nanomolar concentrations S100B is reported to be beneficial, supporting neuronal survival, growth and function [4]. However at higher (micromolar) concentrations there is evidence that S100B can cause apoptosis in neurons and has effects similar to a pro-inflammatory cytokine on astrocytes and microglia [4]. S100B mediates this response through interaction with the receptor for advanced glycation end products (RAGE). RAGE is a multiligand cell receptor which upon ligand binding activates NF-κB via different signalling pathways [5,6].

S100B has been shown to be involved in neurodegeneration and brain injury [7] with elevated levels seen in Alzheimer’s Disease, Parkinson's Disease, Down syndrome and stroke patients [8–10] and it may act as a damage associated molecular pattern (DAMP) protein. S100B has also been associated with chronic inflammation for example in rheumatoid arthritis, diabetes and cystic fibrosis [11]. However, it is not clear whether S100B as a DAMP has a fundamental role as a pro-inflammatory mediator, inducing or exacerbating inflammation in these situations or whether it may play a role in dampening inflammation [12].

There is evidence to suggest that inflammation may be enhanced in these conditions by the action of S100B on macrophages/microglia. *In vitro* studies on microglia cultured from murine BV-2 microglial cell lines have suggested that excessive production of S100B by astrocytes might lead to production of TNF-α, IL-1β, NO and COX-2 by microglia and subsequent enhanced inflammation [6,13]. S100B has also been shown to have a pro-inflammatory effect on the J774 macrophage cell line, for example, stimulating nitric oxide production, inducible nitric oxide synthase (iNOS) protein transcription and TNF-α production in a concentration-dependent manner [14]. However, the influence of S100B on production of other pro-inflammatory cytokines by macrophages, and in primary macrophages has not been studied.

The aim of the study was to clarify the involvement of S100B in inflammation and determine whether its influence is likely to be via macrophages. We have therefore examined its effects *in vitro*, on both a macrophage cell line (RAW 264.7) and on primary macrophages, and also *in vivo*, in a model of retinal inflammatory disease in which pathogenesis is highly dependent on macrophage infiltration (Experimental Autoimmune Uveoretinitis, EAU). S100B has long been known to be present within the eye particularly associated with Mϋller cells [15–17] and is involved in signal transduction in the photoreceptor-bipolar cell region [18]. RAGE has also been shown to be present in the retina and expression is increased in EAU [19].

*In vitro* studies showed that S100B had direct effects on macrophages, enhancing CCL22 and IL-1β expression in particular. EAU disease severity was shown to be reduced in mice in which S100B was deleted and this was related to a reduction in IL-1β and CCL22 expression in the retina. This suggests that S100B may play an active role in enhancing inflammation via its action on macrophages.

## Materials and Methods

### Animals

Animal studies were conducted under a project licence granted by the UK Home Office according to the Animals Scientific Procedures Act 1986. The project was also subject to the University of Aberdeen’s Ethical Review Process and was approved by its Animal Welfare and Ethical Review Body, in accordance with the University Code of Practice for Research Involving the Use of Animals. C57BL/6 wild type (WT) control mice and *S100B* knockout (S100B KO) mice on a C57BL/6 background were supplied by the Medical Research Facility, University of Aberdeen. S100B KO mice were originally established and obtained from the RIKEN Brain Science Institute, Japan [20]. A breeding colony was established in the Medical Research Facility, University of Aberdeen (UK). To confirm the absence of S100B, genotyping was routinely done using PCR as previously described by Nishiyama *et al*., 2002 [20]. Mice were gender and aged matched and used between 8 and 12 weeks old.

### Macrophage cell culture and S100B treatment

To determine the effect of S100B on macrophages, a murine macrophage cell line RAW 264.7 (ATCC, Manassas, USA) was cultured in DMEM plus 10% FCS and 1% penicillin and streptomycin at 37°C 5% CO_2_. Primary macrophages were also cultured from bone marrow (BMDM) of C57BL/6 mice, aged between 8 and 12 weeks. Tibias and femurs were taken and excess tissue removed before sterilizing in 70% ethanol and rinsing in PBS. Bone marrow was flushed out using DMEM/F12 and cells were grown in 6 well plates in DMEM/F12, plus 10% FCS and 1% penicillin and streptomycin, containing 15% L929 conditioned DMEM. After 6 days of culture the cells were characterised as bone marrow derived macrophages by flow cytometry showing high expression of F4/80 (87.4±1.82%; n = 10, ±SEM), CD11b (94±0.35%; n = 10, ±SEM) and low expression of GR1 LY6C (6.63±1.04%; n = 10, ±SEM) and FLT3 (0.626±0.20%; n = 10, ±SEM). When the RAW 264.7 cells reached 80% confluence or on day 6 of primary bone marrow culture, where cells were also approximately 80% confluent, the media was replaced with serum-free media overnight before addition of bovine S100B protein (Sigma Aldrich, Dorset, UK).

The S100B preparation was filtered (0.22 μm) and underwent endotoxin removal treatment using endotoxin removal beads according to the manufacturer’s protocol (Miltenyi Biotec, Bisley, Surrey, UK). LAL endotoxin test (ToxinSensor,GenScript. NJ, USA) confirmed that the endotoxin levels in the S100B protein preparation were minimal at < 6 pg/ml following this treatment. S100B was heated to 95°C for 15 min and used as a control to confirm no endotoxin contamination.

### Cell viability

To determine whether 2 μM S100B was influencing cell viability, morphology was checked and an acid phosphatase cell viability assay done. RAW 264.7 or BMDM were treated with S100B for 24 h. Cells were imaged using a Widefield Ziess Observer microscope (Carl Zeiss, Oberkochen, Germany) and an A1R confocal microscope (Nikon Instruments BV Europe, Amsterdam, Netherlands). For acid phosphatase assay, cells were washed in PBS and incubated with 100 μl of substrate, sodium acetate buffer containing 5 mM p-nitrophenyl phosphate substrate (Sigma-Aldrich, UK). One substrate tablet was dissolved in 2.4 ml sodium acetate buffer (0.1 M sodium acetate and 0.1% Triton). Cells were incubated for 2 h at 37°C. After the incubation period the reaction was stopped by adding 25 μl of 0.1 M sodium hydroxide. The plate was read at 405 nm in a microplate reader (Dynatech MR5000, Dynex Technologies, UK).

### RAGE expression

RNA was extracted from RAW 264.7 and BMDM cells using TRIzol reagent (Invitrogen, Paisley, UK) following manufacturer’s instructions. RNA was quantified (NanoDrop 1000, ThermoScientific, Wilmington, DE, USA) and cDNA generated using superscript II (Invitrogen Paisley, UK) according to manufacturer’s instructions with 2.0 μg total RNA in each reaction and oligo-(dT) priming. The reverse transcription was performed at 42°C for 50 min and 95°C for 10 min. PCR was in a 25 μl total volume reaction mixture containing 12.5 μl Go Taq DNA polymerase reaction buffer (Promega, Southampton UK), 9.0 μl nuclease free water, 0.4 μM forward primer, 0.4 μM reverse primer and 1.5 μl cDNA template. RAGE primers were forward primer, 5’-CAGCATCAGGGTCACAGAAA-3’, and reverse primer, 5’-CTGGTTGGAGAAGGAAGTGC-3’. β-Actin was used as a reference gene, using forward primer, 5’-TGTGATGGTGGGAATGGGTCA-3’, and reverse primer, 5’-TTTGATGTCACGCACGATTTCC-3’. Primers were designed to be intron spanning using the NCBI and primer 3 software. The PCR program consisted of, 1 cycle of denaturation at 95°C for 3 min followed by 30 cycles of 95°C for 30 s, 63°C for 1 min and 72°C for 5 min before a final extension at 72°C for 5 min. The amplified products were analysed by gel electrophoresis on a 1.8% agarose gel containing ethidium bromide. PCR products were visualised under UV light. The resulting cDNA product was visualised at approximately 350 bp for RAGE and 500 bp for β-Actin.

### Western blot

To confirm RAGE protein expression, western blot was carried out on BMDM. BMDM were collected, washed with PBS, centrifuged and re-suspended with 100 μl of 1% NP-40 PBS lysis buffer with the addition of protease inhibitor (cOmplete Protease Inhibitor Cocktail tablets, Roche Products Ltd, Welwyn Garden City, UK) and vortexed and placed on ice for 10 min before a further vortex for 20 s. The samples were then centrifuged at 162 g for 15 min and supernatant removed and stored at -80°C. Protein concentration for the supernatant was determined using BCA protein assay (Pierce BCA Protein assay kit, ThermoScientific). Approximately 60 μg in LDS sample buffer without reducing agents (NuPAGE LDS sample buffer, Life Technologies, Thermo Fisher Scientific) was applied to a non-reduced 12.5% PAGE gel. Mouse lung tissue was homogenised by passing the tissue through a 25G needle and syringe in 1ml NP-40 PBS lysis buffer with protease inhibitor and then treated as for the BMDM and used as a positive control. To identify protein band size, 8 μl MagicMark XP Western Protein Standard (Life Technologies) was also loaded onto the gel.

Proteins were transferred to a PVDF membrane and blocked in 5% non-fat dry milk (AppliChem, Darmstadt, Germany) in 0.1% Tween in PBS for 1 h followed by incubation with 2 μg/ml of Rat-Anti-Mouse RAGE monoclonal antibody (R&D Systems, Abingdon, UK) for 1 h at room temperature. The membrane was then washed 3 x 5 min with 0.1% Tween PBS and incubated with a HRP-conjugated goat anti-rat secondary antibody (1 in 10000, Santa Cruz Biotechnology, Inc, Heidelberg, Germany) for 1 h at room temperature. The membrane was washed again in PBS Tween as previously with an additional 15 min wash. Protein bands were visualised by enhanced chemiluminesence detection system (Pierce ECL Western Blotting Substrate, ThermoScientific) by mixing 1 ml ECL reagents 1 and 2 for 1 min and reading on a luminescent image analyser (ImageQuant LAS-4000 Mini, GE Healthcare Europe, Freiburg, Germany).

### Pro-inflammatory cytokine and chemokine PCR Array

To compare pro-inflammatory cytokine and chemokine gene expression in RAW 264.7 cells cultured for 6 h with or without 2 μM S100B, RNA was extracted using an RNeasy Micro kit (Qiagen, Manchester, UK Ltd) according to manufacturer’s instructions. RNA quality was checked using an Agilent RNA 6000 Nano kit (Agilent Technologies Ltd, Wokingham, Berkshire, UK) and only RNA samples showing clear 18S and 28S peaks, with the 28S:18S ratio higher than 2 were accepted for analysis [21]. RNA was also extracted from retinas from S100B KO or WT mice with EAU. Retinas were removed from mice which had been carefully matched for disease level using TEFI grading and RNA was similarly prepared and checked as described for the RAW 264.7 cells.

From the total RNA extracted, 1 μg was used in cDNA synthesis, using RT^2^ first strand kit (Qiagen, Manchester, UK Ltd). The cDNA was then used in a 96 well RT^2^ Profiler PCR Array for inflammatory cytokines and chemokines (SABiosciences, Qiagen, Manchester UK Ltd) all following manufacturer instructions. A Light Cycler 480 (Roche) was used for the PCR analysis. Cycle threshold values (C_T_) were converted into fold change using Qiagen data analysis software. The RT^2^ Profiler PCR Array for inflammatory cytokines and chemokines has been shown to be a robust method for this type of comparison and to give a good indication of the cytokines and chemokines which warrant further study at the protein level [22,23].

### Real-time PCR to confirm CCL22 and IL-1β gene expression

To confirm the PCR array results from RAW 264.7 cells, which identified *CCL22* and *IL-1β* as responding to S100B, quantitative real-time PCR was done and the time and dose response to S100B determined. RNA was extracted and quantified and cDNA generated as described above. Real-time PCR was set up with 5 μl SYBR Green 2x concentration master mix (Roche), 1 μl forward primer (5 μM), 1 μl reverse primer (5 μM) and 3 μl cDNA and analysed using a Light Cycler 480 (Roche). Primer efficiency was determined by preparation of a standard curve (10 fold) using pooled cDNA, and primers with an efficiency of 1.85–2 were accepted for use. The primer sequences used were designed by Roche universal probe library to be intron spanning and were for CCL22, forward 5’-TCTTGCTGTGGCAATTCAGA-3’ and reverse 5’-gagggtgacggatgtagtcc-3’; and for IL-1β, forward 5’-agttgacggaccccaaaag-3’ and reverse 5’-agctggatgctctcatcagg-3’. Cycle threshold values were normalised to GAPDH gene expression using GAPDH primers, forward 5’-gggttcctataaatacggactgc-3’ and reverse 5’- ccattttgtctacgggacga-3’. The PCR program was 95°C for 5 min followed by 40 cycles of 95°C for 10 s, 60°C for 10 s and 72°C for 10 s. Results are expressed as relative fold change as calculated by the delta delta C_T_ method [24].

### Analysis of CCL22 production by ELISA

To confirm that S100B increases CCL22 production by RAW 264.7 macrophages or BMDM, supernatant was collected after 24 h incubation with or without S100B, centrifuged at 167 g for 5 min at 4°C and stored at -80°C. Supernatants were analysed for CCL22 using a DuoSet ELISA kit (R&D Systems) as recommended by the manufacturer.

### Analysis of IL-1β pro-form production by flow cytometry

For studies on IL-1β pro-form production, RAW 264.7 cells or BMDM were treated with 2 μM S100B and 5 μg/ml Brefeldin A for 4 h (Sigma Aldrich, UK). Cells were harvested in 2% FCS in PBS for flow cytometric analysis of intracellular pro-IL-1β. Cells were incubated on ice for 20 min with Fc block (4 μg of CD16/CD36, BD Biosciences, Oxford, UK) before surface staining with PerCP-CY5.5 rat monoclonal anti-mouse CD11b (BD BioSciences, 0.5 μl of 200 μg/ml). Cells were then fixed and permeabilised in BD BioSciences fixative and permeabilisation buffer, following manufacturer guidelines. PE-conjugated rat anti-mouse IL-1β pro-form (eBioscience Hatfield, UK at 0.06 μg per test in permeabilsation buffer) was added and incubated at room temperature for 30 min in the dark. Cell suspensions were then washed twice in permeabilisation buffer, mixing before each wash, with a final wash in FACS buffer (2% v/v FCS in PBS containing 2% w/v sodium azide). Cells were re-suspended in 300 μl FACS buffer before data acquisition on the LSR II flow cytometer (BD BioSciences). Analysis of FACS data was carried out using FlowJo Software, (Treestar, OR, USA).

### EAU induction

EAU was induced using a standard method [25]. Both S100B KO and C57BL/6 mice were immunised subcutaneously in 2 thighs (50 μl/thigh) with a total concentration of 500 μg per mouse retinol binding protein-3 peptide 1–20 (RBP-3_1−20_; GPTHLFQPSLVLDMAKVLLD; New England Peptide LLC, MA, USA) emulsified with Complete Freunds Adjuvant (CFA-H37Ra, BD Biosciences,) containing additional 2.5 mg/ml *Mycobacterium tuberculosis* (1:1 v/v, BD Biosciences). An additional 100 μl, 10 μg/ml *Bordetella pertussis* toxin was administered by intraperitoneal injection. Mice were sacrificed by CO_2_ asphyxiation and cardiac puncture and eyes collected.

### EAU clinical grading and histological scoring

Disease progression was followed using Topical Endoscope Fundal Imaging (TEFI) at day 15, day 21 and day 24 post peptide immunization (pi) to compare S100B KO and C57BL/6 WT mice. Mice were anaesthetised, pupils dilated using 1% (w/v) tropicamide and 2.5% (w/v) phenylephrine hydrochloride (Bausch & Lomb Minims, Chauvin Pharmaceuticals Ltd, London, UK), and Viscotear liquid gel (Novartis Pharmaceuticals, Frimley, UK) was applied to each eye to provide good endoscope contact to cornea and avoid eyes drying. Fundus images obtained were clinically graded on a scale 0–4 by scoring changes in retinal infiltrate and lesions, retinal vessels and clarity of the optic disc according to Xu *et al*.,2008 [26]. At approximately peak disease, day 24 pi, mice were culled and eyes were snap frozen in OCT compound (Tissue-Tek, Agar Scientific, Essex, UK). Eyes were serially sectioned at 6 μm and sections fixed in acetone, haematoxylin stained and dehydrated in ethanol. Sections were used to grade disease based on cell infiltration and retinal structure, as described in Copland *et al*., 2008 [27]. Sections were masked and graded using 3 sections at each of 3 spaced intervals through the eye.

### Immunohistochemistry for S100B in retina

To examine the expression of S100B in the retina, immunohistochemistry was carried out using a Nova Red peroxidase substrate kit prepared as per manufacturer instructions (Vector Laboratories, CA). Sections were fixed in 100% ethanol for 5 min before being rehydrated in PBS and blocked with 10% normal rabbit serum for 30 min. F(ab’)_2_ Fab polyclonal anti-mouse IgG (Serotec) was applied for 30 min before incubation with anti-mouse S100B primary antibody (Sigma, UK) at a 1 in 500 dilution in 1% normal swine serum for 1 h at room temperature followed by washing in PBS. Sections were then incubated with the secondary antibody, 1 in 200 dilution, for 1 h before washing in PBS. ABC elite peroxidase (Vector PK6100, Vector Labs, Ca, USA) was applied for 30 min at room temperature, slides washed in PBS as previously, then the Nova Red substrate (SK4800) was applied. The substrate reaction was stopped after 30 s by washing in water. Sections were counterstained with haematoxylin before being dehydrated and mounted in DPX (Sigma).

### Immunohistochemistry for macrophages

Sections were stained for macrophages with MOMA-2 antibody (purified rat anti-mouse polyclonal antibody; AbDSerotec, Oxfordshire, UK) using a 1 in 25 dilution. The secondary antibody was used at a 1 in 100 dilution (biotinylated anti-rat IgG; Vector Labs). In control staining, primary antibody was replaced with tris-buffered saline. Sections were masked and total stained and unstained infiltrating cells present across the retinal layers, or within the rod outer segments were counted using a graticule to count cells in an exact area (1.5 mm^2^) in 3 sections at 3 spaced intervals through the eye. The average count for the sections was calculated for each eye.

### Statistics

Statistical analysis was carried out using Mann-Whitney non-parametric test, Student’s unpaired T Test and ANOVA.

## Results

### S100B up-regulates pro-inflammatory gene transcription in macrophages

**Fig 1:**
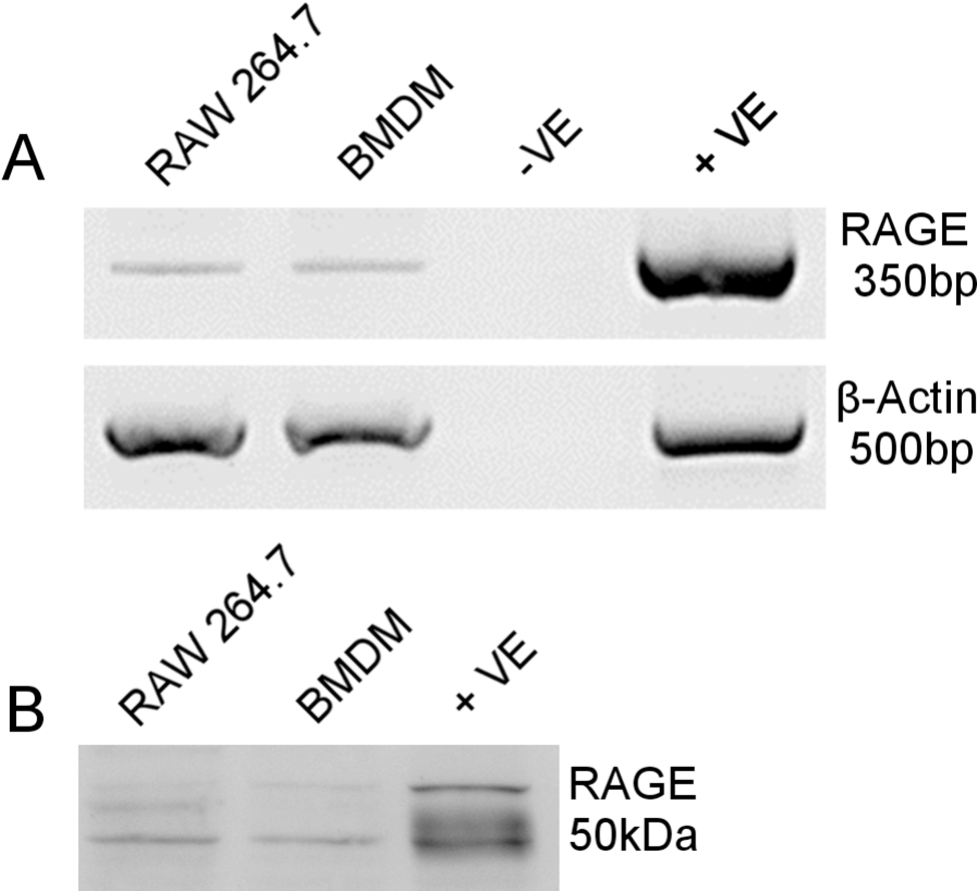
RAGE gene expression of RAW 264.7 macrophages and BMDM. A) Agarose gel electrophoresis of RT-PCR products indicates RAGE band at expected band size of 350 bp and β-actin as reference gene with band size 500 bp. Mouse lung sample used as positive control (+ve), with negative control (-ve) containing water in place of cDNA. B) Western blot for RAGE showing predicted 50 KDa band for RAW264.7 macrophages and BMDM and for positive control (+ve) mouse lung.

To investigate the direct effect of S100 on macrophages we first used the mouse macrophage cell line, RAW 264.7. RAGE mRNA expression by RAW 264.7 and BMDM cells was confirmed by routine RT-PCR (Fig 1A). RAGE protein in RAW cells (previously shown[28]) and BMDM was confirmed by western blot (Fig 1B). The effect of S100B on RAW 264.7 macrophages in terms of inflammatory cytokine and chemokine response was examined at a transcriptional level using real-time PCR array analysis. Unlike previous studies [14] which have only focused on a limited selection of pro-inflammatory cytokines this allows a wide range of pro-inflammatory cytokines, chemokines and their receptors to be examined.

**Fig 2:**
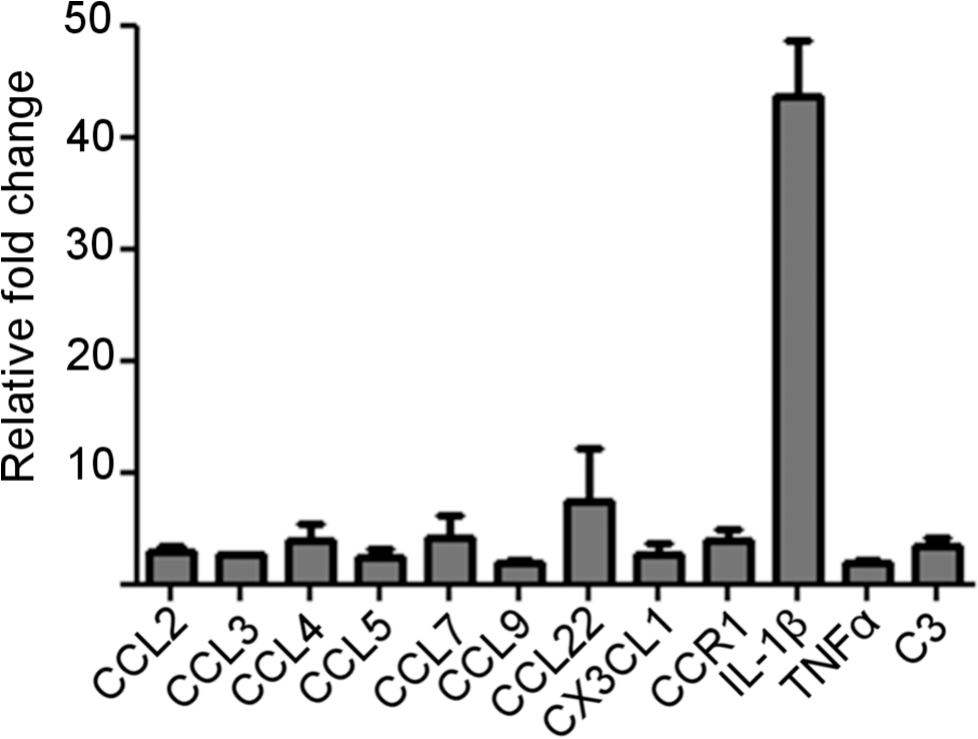
PCR array for inflammatory cytokines, chemokines and receptors on RAW 264.7 macrophages treated with or without S100B (2 μM for 6 h). Graph shows relative fold change in response to S100B for those genes with >2 fold change after S100B treatment (2^-ΔΔCT^). Fold regulation normalised to GAPDH. Mean of two individual experiments each with 3 pooled samples.

RNA extracted from cells was confirmed to be high quality before use in the array. Real-time PCR cycle threshold (C_T_) value, (the value where there is a significant detectable increase in fluorescence above the baseline value), was calculated using the second derivative max, and allowed fold change calculations to be made. Each array provided quality control checks for PCR reproducibility, reverse transcription and genomic DNA contamination. Five reference genes were available to select the optimum gene normalisation. All samples were normalised to GAPDH and C_T_ values greater than 35 were not reported. RNA samples from RAW 264.7 cells treated with or without S100B and meeting PCR array quality control standards were reverse transcribed, analysed using the PCR array and normalised to GAPDH. The PCR array showed an overall up-regulation of pro-inflammatory mediators in S100B treated compared to untreated cells (Fig 2). In particular, expression of *CCL22* and *IL-1β* was increased over seven-fold in response to S100B treatment.

**Fig 3:**
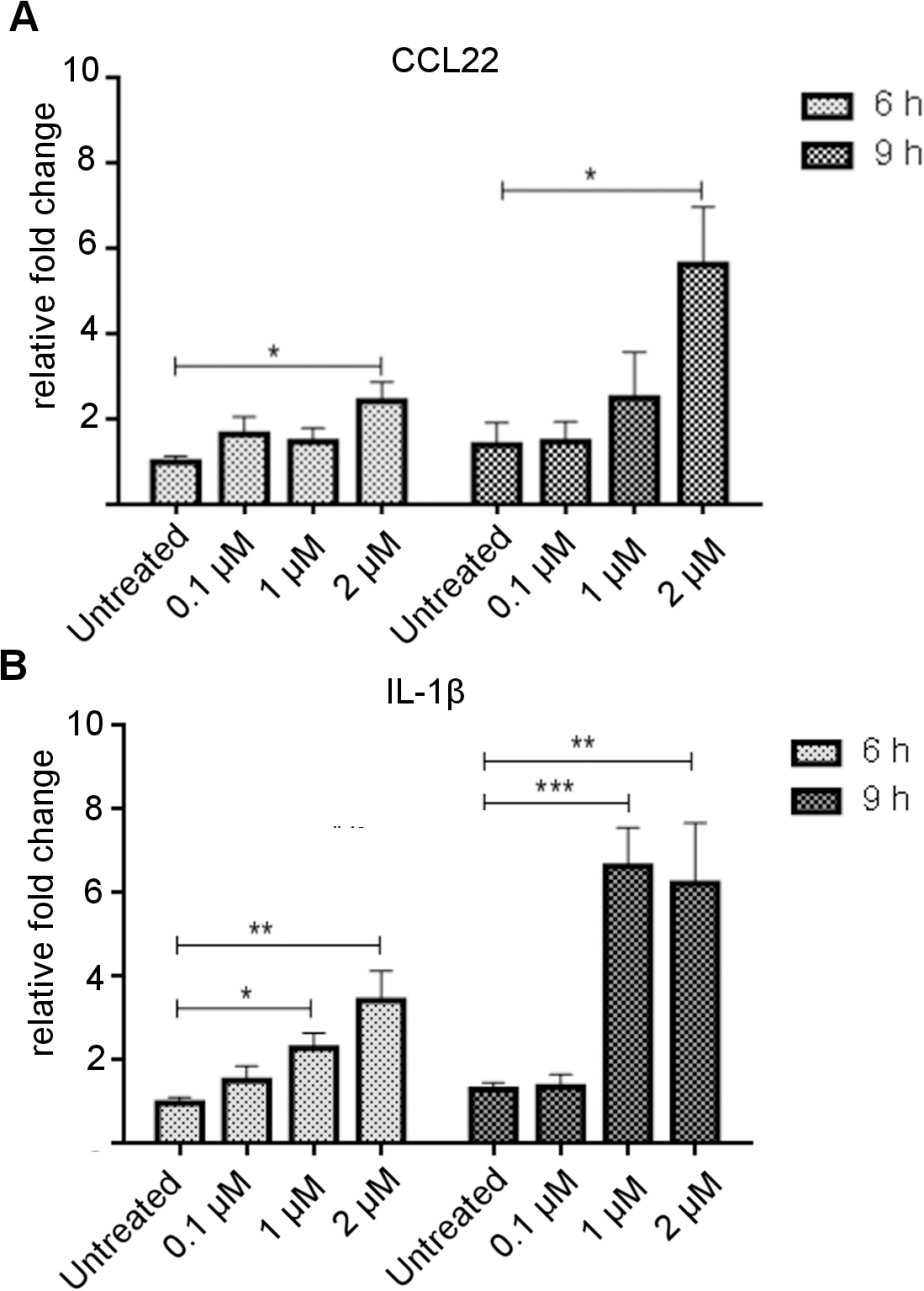
Confirmation of increased CCL22 and IL1β expression in RAW 264.7 macrophages in response to increasing dose of S100B. Relative fold increase in mRNA expression for CCL22 (A) and IL-1β (B) determined by real-time PCR analysis. Error bars indicate SEM. n = 6. * *P*<0.05, **P<0.005, ***P≤0.001 (ANOVA).

Gene expression for CCL22 and IL-1β was investigated further using real-time PCR and a range of S100B doses and incubation times. S100B was confirmed to up-regulate CCL22 and IL-1β gene expression (Fig 3). CCL22 showed its highest fold increase in mRNA expression (4.2 fold, *P* = 0.0131) in response to incubation of RAW 264.7 cells with 2 μM S100B for 9 h (Fig 3A). There was also a significant increase (*P* = 0.0170) after 6 h incubation at this concentration. *IL-1β* showed a significant increase in RAW 264.7 cells compared to untreated cells with 1 μM and 2 μM S100B (Fig 3B).

### S100B increases CCL22 production in macrophages

**Fig 4:**
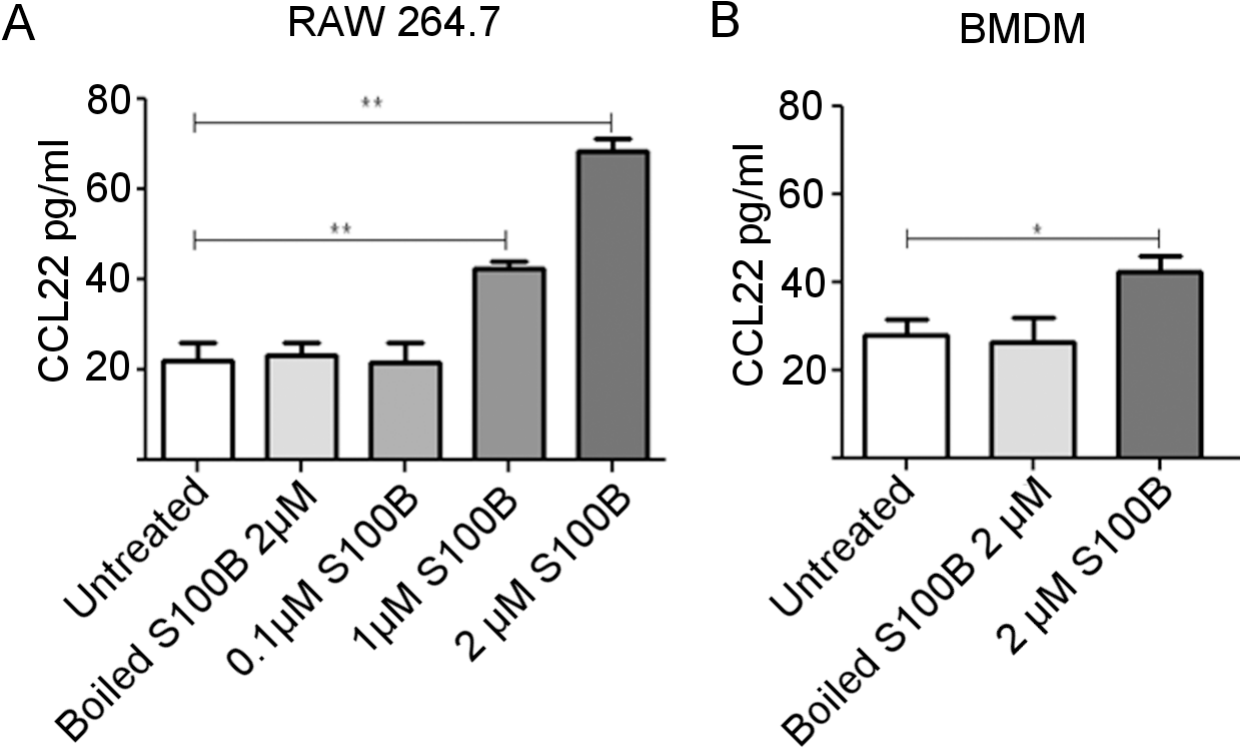
CCL22 production by macrophages in response to S100B treatment for 24 h as determined by ELISA on supernatants. CCL22 production by RAW 264.7 cells (A) and BMDM cells (B). There was a significant increase in CCL22 with 1 μM S100B and 2 μM S100B in RAW 264.7 macrophage cells and with 2 μM in BMDM cells compared to untreated control cultures. Representative of 3 individual experiments. *P<0.05, **P<0.001 (ANOVA). Error bars indicate SEM n = 3.

**Fig 5:**
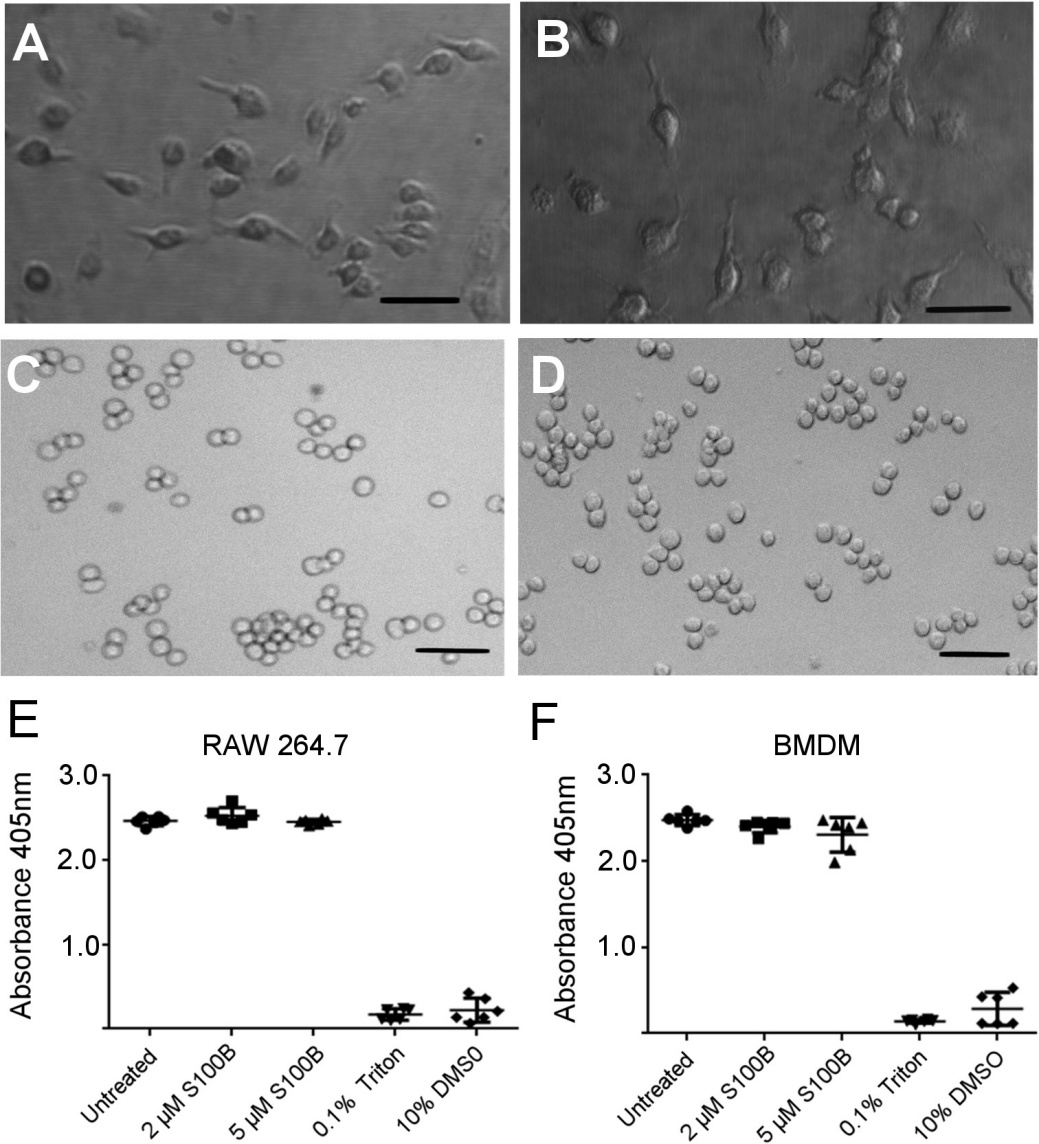
Treatment of macrophages with S100B does not reduce cell viability as indicated by cell morphology and acid phosphatase assay. Images to show BMDM untreated (A) and treated with 2 μM S100B for 24 h (B) and RAW 264.7 untreated (C) and treated with 2 μM S100B for 24 h (D). Scale bars = 50 μM. Acid phosphatase cell viability assay on RAW 264.7 untreated or treated with 2μM or 5 μM S100B for 24 h (E) and on BMDM (F). No significant difference in viability following treatment with 2μM S100B for 24 h. Triton (0.1%) and DMSO (10%) as positive controls for the assay. Error bars indicate SEM n = 6.

To determine whether S100B increased CCL22 production as well as mRNA expression, CCL22 in cell supernatant was analysed by ELISA (Fig 4). S100B increased CCL22 production by RAW 264.7 cells in a concentration dependent manner with a significant increase in CCL22 in the presence of 2 μM (*P* = 0.0032) and 1 μM S100B (*P* = 0.0078) compared to untreated control cultures (Fig 4A). This was also confirmed in primary macrophages, BMDM, where a significant increase in CCL22 production was observed at 2 μM S100B treatment (Fig 4B). No increase in CCL22 production occurred with cells incubated with S100B that had been boiled. To check that these responses to S100B were not related to the effects of S100B on cell viability, cell morphology was monitored and viability was measured by acid phosphatase assay following treatment of cells with 2 μM or 5 μM S100B for 24 h. There were no morphological differences and there was no significant difference in cell viability following treatment of either RAW 264.7 cells or BMDM with 2 μM or 5 μM S100B for 24 h (Fig 5).

### S100B increases pro-IL-1β production by macrophages

**Fig 6:**
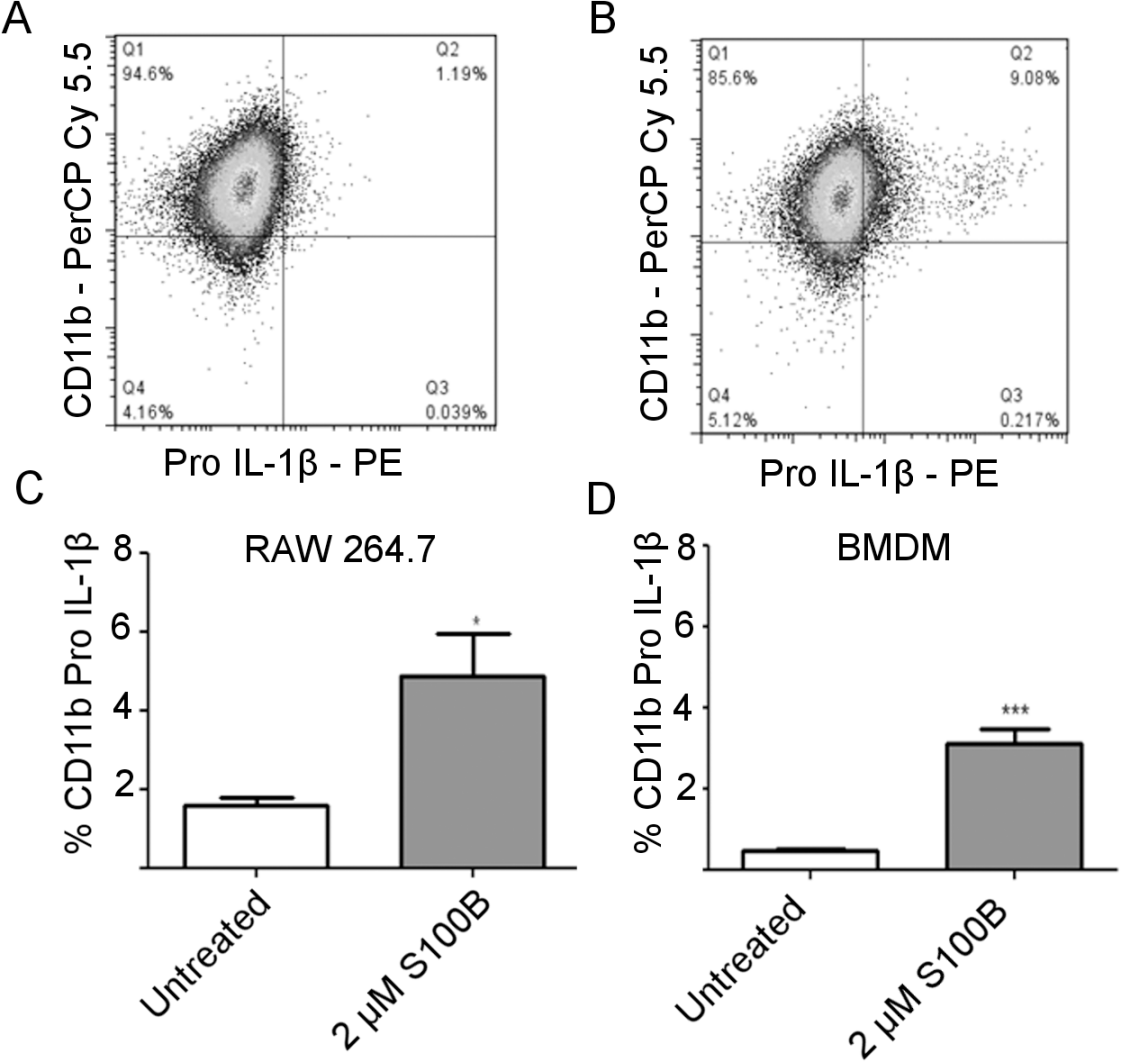
S100B treatment of macrophages results in increased pro-IL1β expression. Representative dot plots showing flow cytometric analysis of RAW 264.7 cells using anti-CD11b (PerCP-Cy 5.5) to gate macrophages and anti-pro-IL1β (PE) to detect intracellular staining in untreated cells (**A**) and in cells treated with 2μM S100B (**B**). Significant increase in the percentage of CD11b positive cells expressing pro-IL-1β in response to S100B is shown graphically in both RAW 264.7 macrophage cells (*P<0.05) (**C**) and BMDM (***P<0.001) (**D**). Error bars indicate SEM n = 4.

To confirm that S100B up-regulated transcription of IL-1β results in protein translation, flow cytometry was used to detect pro-IL-1β after treatment of RAW 264.7 cells and BMDM with 2 μM S100B for 4 h. Cells were gated using CD11b expression (Fig 6A and 6B). Compared to untreated control cultures, S100B treated RAW 264.7 cells (Fig 6C) and BMDM (Fig 6D) showed a significant increase in the percentage of cells expressing pro-IL-1β (*P* = 0.0338).

### S100B is increased in EAU diseased retinal sections

**Fig 7:**
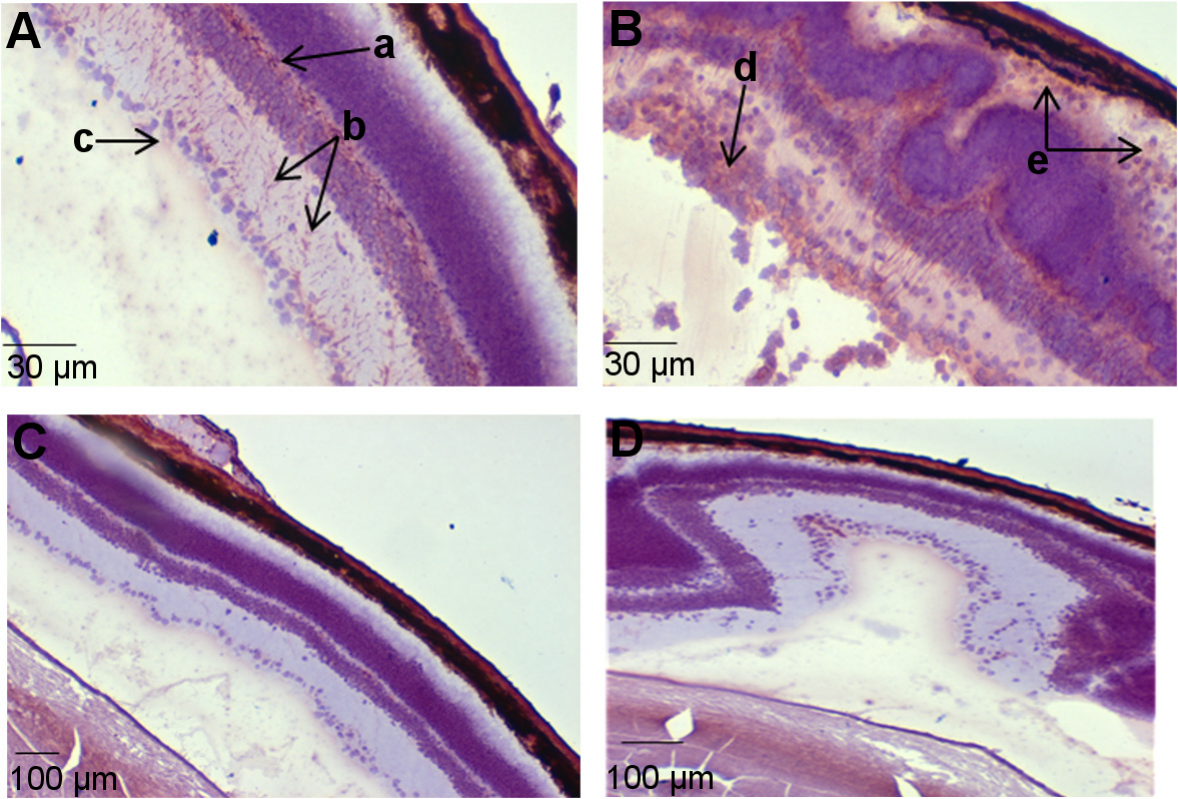
Increase in S100B was detected in retinal sections from WT mice with EAU compared to untreated mice using immunohistochemistry. **(A)** Positive staining was observed in the naïve retina, specifically in the outer plexiform layer (a), inner plexiform layer (b) and retinal ganglion layer (c). Increased staining was observed in EAU diseased sections **(B)** in the retinal ganglion layer (d) and in the rod outer segments where positive staining was located around infiltrating cells (e). No positive staining was observed in control sections, where S100B antibody was replaced with PBS in naïve retina **(C)** or in EAU diseased retina **(D).** Serial sections from naïve and diseased mice taken at spaced intervals throughout the eye.

To investigate the relevance of the pro-inflammatory effect of S100B on macrophages *in vivo* we used a murine model Experimental Autoimmune Uveoretinitis (EAU), in which retinal pathogenesis is highly dependent on macrophage infiltration and activation [29,30]. To determine whether S100B protein levels are up-regulated in the retina in response to EAU induction, immunohistochemistry was carried out on retinal sections taken from mice in which EAU had been induced or untreated mice. Positive staining was observed in the naïve retina (Fig 7), specifically in the retinal ganglion layer and outer plexiform layer which confirms previous studies which have identified S100B present within the retinal layers [31]. An increase in S100B positive staining was observed in EAU diseased sections, specifically in the rod outer segments, where positive staining was located around infiltrating cells, and in the retinal ganglion layer (Fig 7).

### Removal of S100B dampens the inflammatory response in EAU

**Fig 8:**
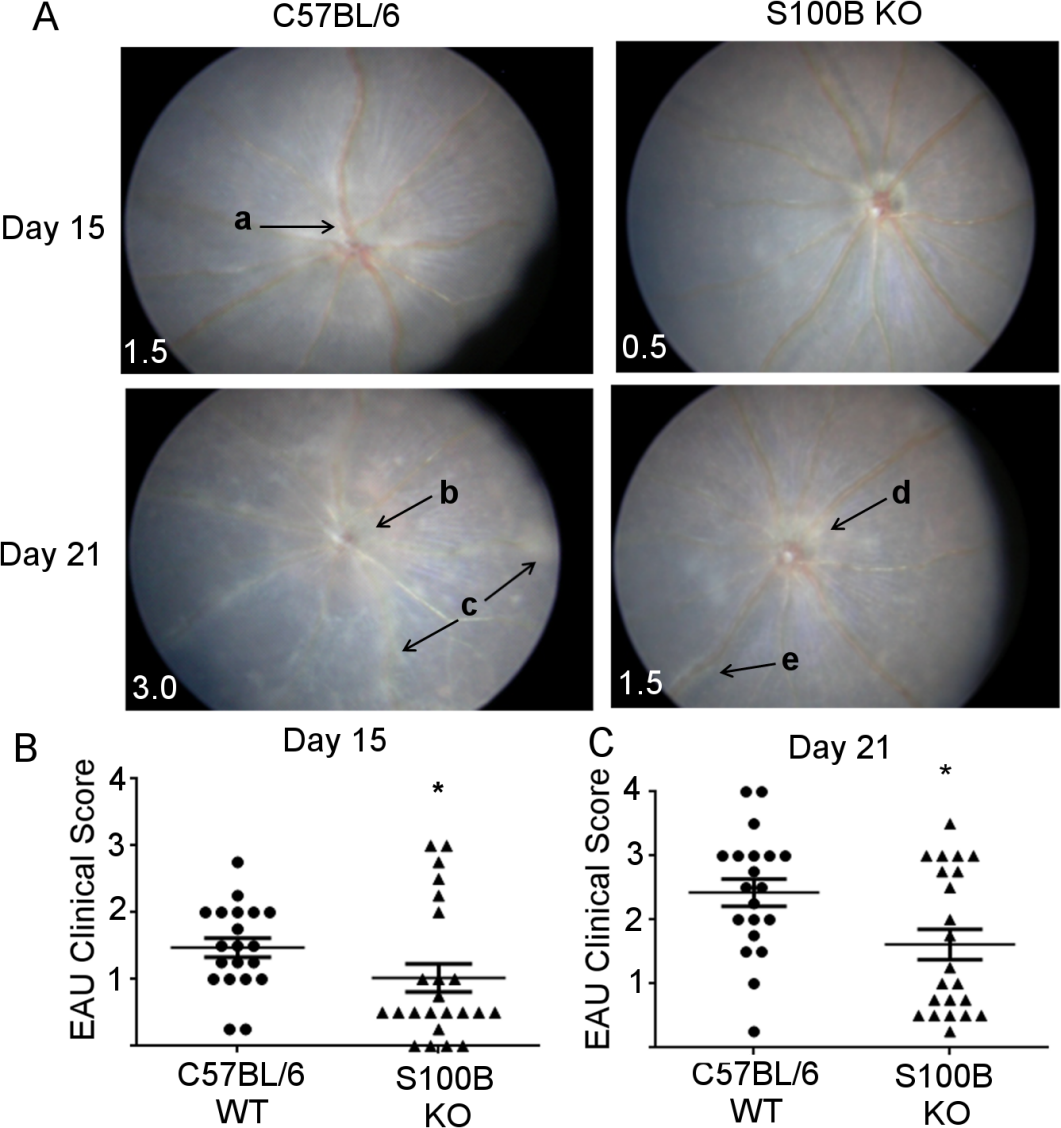
EAU TEFI grading in C57BL/6 and S100B KO mice at day 15 and day 21 pi. **A**, vascular cuffing (a) indicating inflammation in C57BL/6 day 15 pi. Severe inflammation in C57BL/6 at day 21 pi, with swollen, barely visible optic disk (b), cell infiltrates (c), and progression of vascular cuffing. In comparison, S100B KO images showing reduced disease but evidence of inflammation. Optic disk appears swollen day 21 pi (d) with vascular cuffing occurring on both sides of vessels (e). Approximate diameter of eye 3.3 mm. **B**, TEFI clinical grading scores for EAU at day 15 pi and **C,** day 21 pi indicating significantly reduced disease in S100B KO mice (*P<0.05). TEFI scores for individual eyes from the same mouse were averaged and each point represents one animal. Results from two independent experiments were combined.(C57BL/6 n = 18 mice S100B KO n = 22 mice). Error bars indicate SEM.

To determine whether the absence of S100B affects the inflammatory response in EAU, S100B KO and C57BL/6 WT controls were immunised with RBP-3_1−20._ Progression of disease was followed using TEFI at day 15 and day 21 pi (Fig 8A). Fundus images were graded for retinal inflammation (Fig 8B and 8C). By day 15 pi both groups showed signs of inflammation; however, the S100B KO mice showed a significant decrease in EAU grade compared to WT (*P<0.05) (Fig 8B). Inflammation had increased in both strains by day 21 pi but S100B KO mice still had significantly less inflammation (Fig 8C).

**Fig 9:**
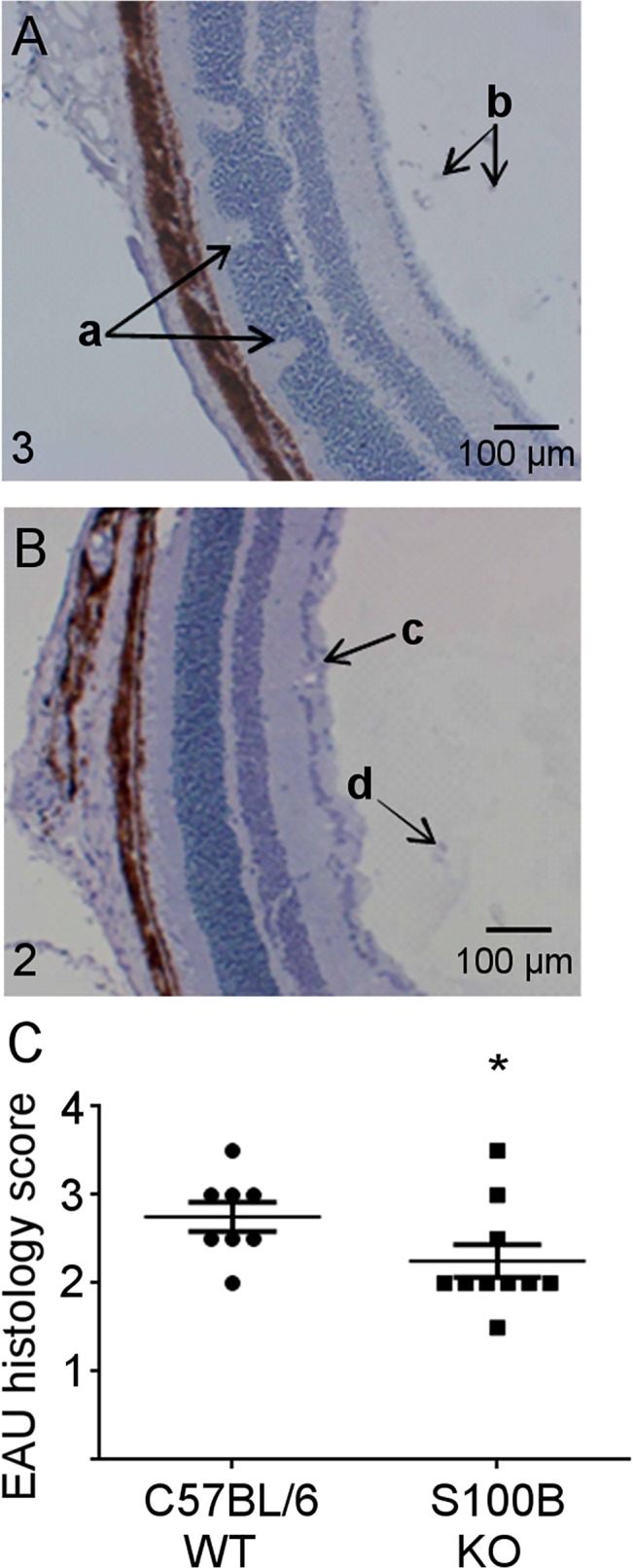
Histological grading of EAU in C57BL/6 WT and S100B KO retinal sections at day 24 pi. Eyes were snap frozen and serially sectioned at 6 μm. Sections were graded for EAU. **A,** section showing EAU disease in C57BL/6 mouse (infiltrative grade 3) with folding of retinal layers (a) and cell infiltrates in the vitreous (b) and retinal layers. **B,** section showing reduced EAU disease (infiltrative grade 2) in S100B KO mice where layers have remained intact, showing cells gathering around vessel wall (c) and infiltrating cells in the vitreous (d). **C**, significantly decreased levels of retinal infiltrating cells were seen in sections from S100B KO mice (*P<0.05). Grading scores for individual eyes from the same mouse were averaged and each point represents one animal. C57BL/6 n = 8 mice, S100B KO n = 10 mice. Error bars indicate SEM.

Eyes were collected at day 24 pi for histological evaluation. Histology sections were graded according to cell infiltration and confirmed TEFI observations showing significantly reduced inflammatory infiltrate in the S100B KO mice compared to WT (Fig 9, *P = <0.05).

**Fig 10:**
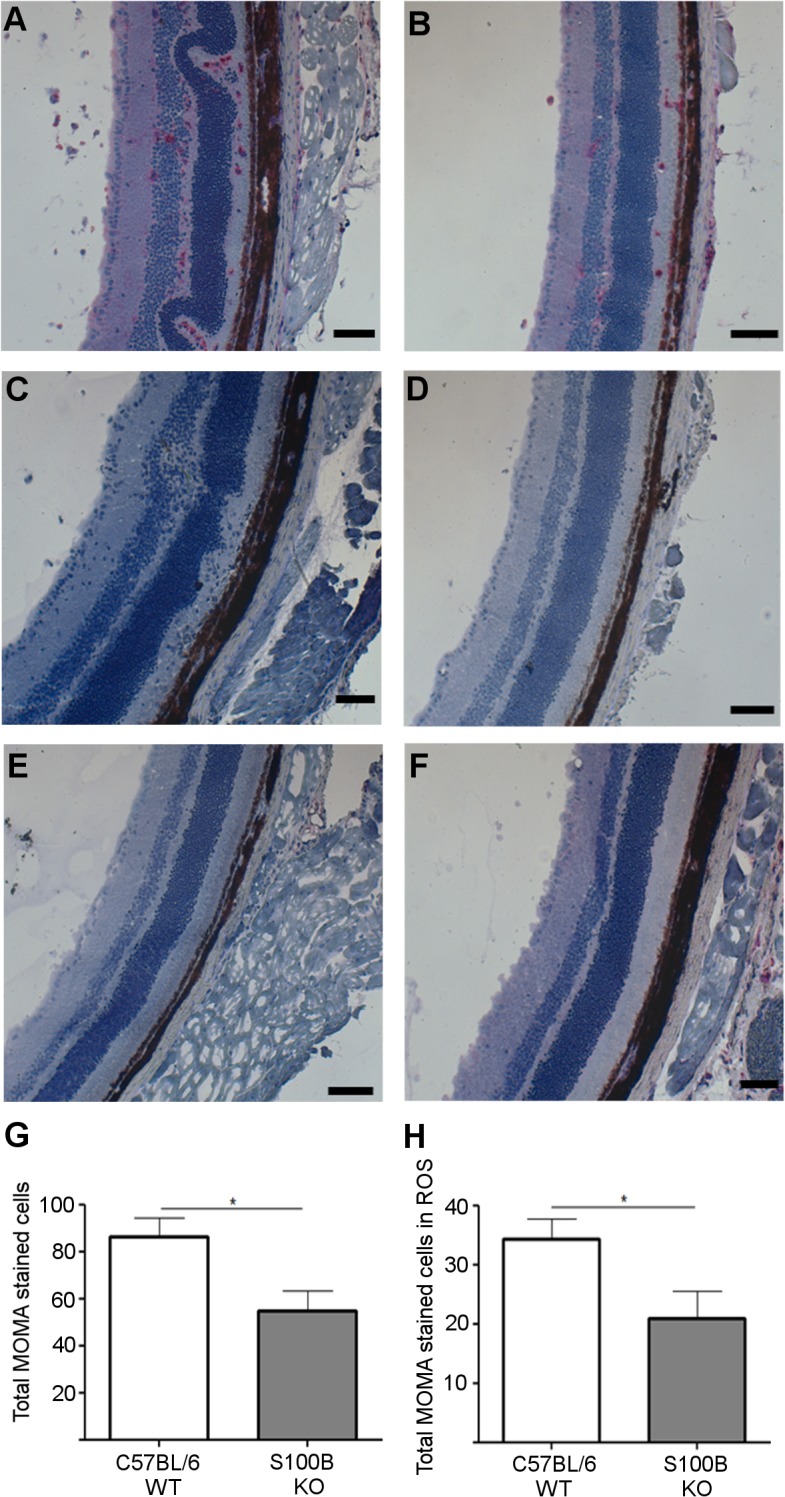
Monocyte/Macrophage infiltration in sections from C57BL/6 and S100B KO mice. **A**, C57BL/6 WT retinal section from mouse with EAU showing cells staining positively for MOMA-2 (red). **B,** positive MOMA-2 staining in S100B KO with EAU was also observed in the rod outer segments and vitreous. No positively stained cells were observed in negative control staining in C57BL/6 mice **(C)** or S100B KO mice with EAU **(D),** where primary antibody was replaced with TBS. No positive staining was observed in naïve C57BL/6 WT **(E)** or S100B KO mice **(F)**. Cells positive for MOMA-2 were counted in a total area of 1.5 mm^2^. An average cell number was calculated from 3 sections taken at spaced intervals through the eye. A significant reduction in positively stained cells was observed in S100B KO mice compared to C57BL/6 WT mice both in the retinal layers overall **(G)** and specifically within the rod outer segments (ROS) **(H).** Error bars indicate SEM; n = 8 randomly selected eyes per genotype. Scale bars indicate 100 μm.

To determine the effect of *S100B* deletion on macrophages in the inflammatory infiltrate the infiltrate was stained using MOMA-2 (Fig 10A–10F). Eyes were serially sectioned and 3 sections at different points through the eye were examined. A graticule was used to count total stained cells within an equivalent area in the retina from S100B KO mice or C57BL/6 WT mice. A significant decrease in macrophage cells was observed in retinal sections taken from the S100B KO mice compared to C57BL/6 WT mice (Fig 10G). A significant reduction in macrophage infiltration specifically in the rod outer segments was also observed (Fig 10H). No positive staining was observed in healthy control retina sections from either C57BL/6 mice (Fig 10E) or S100B KO (Fig 10F).

### CCL22 and IL-1β gene transcription were down-regulated in the diseased retina in S100B KO mice

Real-time PCR was used to compare retinas from WT and S100B KO mice in which the disease was of the same severity, grade 3 as determined by TEFI, and confirmed that CCL22 and IL-1β were reduced specifically in S100B KO mice compared to WT with a fold reduction normalised to GAPDH of 3.58 for CCL22 and 4.63 for IL-1β.

## Discussion

Our aim was to determine whether S100B could influence the production of cytokines and chemokines by macrophages and whether this was likely to be relevant in inflammatory disease in which macrophages are key effector cells such as EAU. Although S100B has previously been shown to have a pro-inflammatory effect on macrophage cell lines [14,32], production of a full range of pro-inflammatory cytokines and chemokines by macrophages in response to S100B has not been investigated and production has not been confirmed in primary macrophages.

The macrophage cell line, RAW 264.7, was used for initial studies and we confirmed the expression of RAGE in these cells and in BMDM. RAGE has also been shown to be expressed by infiltrating macrophages in EAU [19]. PCR array analysis, using an established, commercially prepared PCR microarray [22,23], on RAW 264.7 cells treated with 2 μM S100B for 6 h showed that TNFα was up-regulated, as shown previously [14,32], as well as other cytokines. However the largest increases were seen in IL1β and CCL22.

Rapid up-regulation of IL-1β transcription in macrophages in response to S100B was confirmed by real-time PCR and corresponding up-regulation of intracellular pro-IL-1β production was shown by flow cytometry in RAW 264.7 and for also the first time, to our knowledge, in primary BMDM. Up-regulation of pro-IL-1β does not confirm the presence of the mature protein and its secretion. Complex regulation of the inflammasome is required for IL-1β secretion [33]. It is not possible to show mature IL-1β in RAW 264.7 cells due to absence in these cells of an essential adaptor protein, ASC, needed for caspase 1-mediated IL-1β processing in inflammasomes [34]. Thus, although we are not able to confirm this *in vivo* in this study, we propose that under fully functioning inflammasome conditions *in vivo*, mature IL-1β production by macrophages will be up-regulated by S100B. S100B has previously been seen to promote IL-1β production in primary rat astrocyte cultures, primary rat microglia and a murine microglial cell line BV-2. IL-1β mRNA expression in this microglial cell line was shown to be elevated in the presence of S100B via RAGE [6,35,36]. S100B treatment of a human monocytic cell line, THP-1, has also been reported to result in increased IL-β gene transcription and secretion [37].

IL-1β is a highly pro-inflammatory cytokine and a major source of it in the inflammatory response is the macrophage. Locally it acts to enhance the recruitment of further inflammatory leukocytes [38]. It is a key pro-inflammatory cytokine involved in EAU [39]. Its proposed increased production in response to S100B *in vivo* would therefore be likely to exacerbate inflammatory disease.

S100B up-regulated the expression of CCL22 by RAW 264.7 cells up to 7 fold and up-regulation of protein production was also shown by ELISA with CCL22 secretion increased in response to S100B in both RAW 264.7 and primary BMDM. CCL22 is an important chemoattractant produced by macrophages and DC under inflammatory conditions. Although its production is associated with M2 macrophages and it can recruit Th2 cells and T regulatory cells via its receptor CCR4, indicating a more regulatory role [40], it may also increase pathogenesis. It has been shown to promote tissue injury in bleomycin-induced pulmonary fibrosis in mice by inducing the M1 macrophage phenotype [41]. In addition, CCL22 is elevated in the CSF of patients with MS and both CCL22 and CCR4 have been shown to contribute towards the recruitment and function of inflammatory macrophages and disease development in experimental autoimmune encephalitis (EAE) [42–44]. Enhanced levels of S100B have also been reported in EAE [43]. Increased CCL22 has been reported in EAU [39] and it is possible that as in EAE it is acting to exacerbate disease.

If S100B acts predominantly as a direct pro-inflammatory mediator it should exacerbate inflammation. However, for these *in vitro* experiments on macrophages a relatively high dose 2 μM was shown to be effective. This is consistent with other studies where higher doses of S100B were shown to be most pro-inflammatory [4]. S100B has been shown to be present at these levels in tissue for example in brain damage where it is reported to accumulate in the extracellular space [45] but it is not known whether in other situations it is present at levels which would be likely to influence inflammation. To test the relevance of S100B in inflammatory disease *in vivo* we have investigated its role in retinal inflammation in EAU in which macrophages play a crucial role in defining the severity of the disease and the subsequent damage incurred [30].

Although S100B has been reported to be present within the eye it is unclear whether it is present in sufficient concentration to influence inflammation so initially we examined S100B expression in the retina. We have shown for the first time, to our knowledge, an increase of S100B in the retina of mice with EAU compared to healthy retina. In the eye S100B will be produced by glial cells, astrocytes and Mϋller cells [16,46,47] and will accumulate in the extracellular matrix and there may also be S100B release by damaged cells. Our immunohistochemical staining showed evidence of this. In the brain, where enhanced synthesis of S100B has been shown by reactive astrocytes accumulating around the infarct area after cerebral artery occlusion, it was concluded that in areas of intense S100B staining S100B tissue concentration reaches micromolar concentration albeit temporarily [48]. In our tissue sections there was also evidence of S100B production associated with infiltrating cells. It has been suggested that T cell production of S100B, although less than that of astrocytes, may, when polarized in the immunological synapse, be sufficient to trigger macrophage activation [49]. Recently S100B has been shown to be up-regulated in the eye in response to laser photocoagulation which generated choroidal neovascularisation [50]. Thus it is likely that S100B is present in the retina in EAU at the concentrations we have shown *in vitro* are able to influence macrophage response.

To examine the role of S100B in inflammation we investigated the effect of deletion of S100B on induction of retinal inflammation in EAU. Clinical grading showed that EAU severity was significantly reduced in S100B KO compared to WT mice from day 15 pi. This was confirmed by histological examination at day 24 pi which showed a significant reduction in cell infiltration in S100B KO mice compared to WT. Immunohistochemistry indicated a reduction of macrophages within the retina of S100B KO mice with EAU compared to C57BL/6 WT mice with EAU. Overall reduction in the infiltrate in S100B KO mice is likely to be due to a general reduction in adhesion molecule and chemokine levels for inflammatory cells. IL-1 in particular has a major influence on these factors. Initial T cell and macrophage recruitment to the retina in S100B mice with EAU is unaffected as onset of disease is not delayed. However, macrophages in the retina of S100B KO mice then produce less IL-1 and CCL22 and further inflammatory cell recruitment is reduced. In EAE it has been reported that CCL22 enhances myeloid cell recruitment [44]. Production of S100B during uveitis either by resident cells in the eye or by infiltrating cells is therefore likely to have up-regulated CCL22 and IL-1β in particular resulting in further recruitment of inflammatory cells and exacerbation of disease.

Although average disease severity was reduced in S100B KO mice compared to WT and consequently the overall average levels of CCL22 and IL-1β would be decreased we were interested in comparing levels of CCL22 and IL-1β in mice selected for the same level of disease. Matching disease in WT and in S100B KO mice enabled us to highlight changes that might be more directly related to S100B and its underlying role rather than those which would generally follow from reduced disease severity. PCR analysis of retinas matched for disease severity (Grade 3 TEFI) confirmed reduction of CCL22 and IL-1β in retinas where S100B had been deleted.

Thus we have shown for the first time that S100B is an important component in the development of retinal inflammation. It is likely to be present at levels that may have a direct action on macrophages, the key effector cell in uveoretinitis, up-regulating chemokine/chemokine receptor and cytokine expression, CCL22 and IL-1β in particular. Recently it has been shown that S100B can increase migration of BMDMs [50] and this, in conjunction with any up-regulation of CCL22 and IL-1β, would lead to the recruitment of further cells and an exacerbated inflammatory response. The effect of S100B on infiltrating macrophages will be combined with effects on resident microglia cells in the retina further enhancing this response. The reduction in disease severity in S100B KO mice may also be due to effects on other cell types expressing RAGE such as DC and further experiments will be required to determine the extent to which the reduction in severity in EAU as a result of S100B deletion is directly due to its effects on macrophages, and in particular the role of IL-1β and CCL22. However, it is likely that S100B is able to exacerbate inflammatory disease at least in part through its action on macrophages.
